# Antibody-guided design and identification of CD25-binding small antibody mimetics using mammalian cell surface display

**DOI:** 10.1038/s41598-021-01603-w

**Published:** 2021-11-11

**Authors:** Kyra See, Tetsuya Kadonosono, Kotaro Miyamoto, Takuya Tsubaki, Yumi Ota, Marina Katsumi, Sumoe Ryo, Kazuki Aida, Misa Minegishi, Tatsuhiro Isozaki, Takahiro Kuchimaru, Shinae Kizaka-Kondoh

**Affiliations:** 1 School of Life Science and Technology, Tokyo Institute of Technology, Yokohama, 226-8501 Japan; 2 Center for Molecular Medicine, Jichi Medical University, Tochigi, 329-0498 Japan

**Keywords:** Protein design, Peptides

## Abstract

Small antibody mimetics that contain high-affinity target-binding peptides can be lower cost alternatives to monoclonal antibodies (mAbs). We have recently developed a method to create small antibody mimetics called FLuctuation-regulated Affinity Proteins (FLAPs), which consist of a small protein scaffold with a structurally immobilized target-binding peptide. In this study, to further develop this method, we established a novel screening system for FLAPs called monoclonal antibody-guided peptide identification and engineering (MAGPIE), in which a mAb guides selection in two manners. First, antibody-guided design allows construction of a peptide library that is relatively small in size, but sufficient to identify high-affinity binders in a single selection round. Second, in antibody-guided screening, the fluorescently labeled mAb is used to select mammalian cells that display FLAP candidates with high affinity for the target using fluorescence-activated cell sorting. We demonstrate the reliability and efficacy of MAGPIE using daclizumab, a mAb against human interleukin-2 receptor alpha chain (CD25). Three FLAPs identified by MAGPIE bound CD25 with dissociation constants of approximately 30 nM as measured by biolayer interferometry without undergoing affinity maturation. MAGPIE can be broadly adapted to any mAb to develop small antibody mimetics.

## Introduction

Identification of target-binding molecules is important in both the development of novel drugs and the improvement of available drugs. In particular, antibody mimetics, small proteins and peptides that bind to the same target antigen as therapeutic monoclonal antibodies (mAbs), are highly desirable because of cheaper manufacturing requirements and potentially improved tissue penetration compared with mAbs^1^. To identify and engineer such molecules, several molecular display systems have been developed in which genetically encoded library molecules are fused to endogenous proteins and displayed on the surface of phages^2^ or the cell surface of bacteria^3^, yeasts^4^, or mammalian cells^5^. While phage display systems are a popular option to screen libraries of both antibody fragments^6^ and peptides^7^, cell display systems are also often used because of their compatibility with fluorescence-activated cell sorting (FACS), which allows rapid selection of cells that express high-affinity binders on their surface.

Mammalian display systems have been used to identify a wide range of molecules that include peptides^8,9^, antibody fragments^10–12^, and even whole antibodies^13^. However, similar to other molecular display systems, several rounds of selection are usually required to concentrate high-affinity binders from a large library. Furthermore, screening libraries using mammalian display generally involves expression of the library on the cell surface, followed by incubation of the cells with the labeled target protein prior to cell sorting to identify cells that express target-binding molecules^14^. Because this largely limits the range of target proteins to soluble proteins, screening against membrane proteins may be problematic. To overcome this limitation, a recent report has described the development of a mammalian display system using cells that have been engineered to express both the target antigen and tagged library. The former is expressed on the cell surface while the latter is secreted into solution, which results in “self-labeling” of the cells upon binding of the tagged molecules to the target protein^15^. However, a potential issue with this system is the occurrence of cross-labeling or binding of secreted molecules to the surface of another cell.

Previously, we developed a design strategy to create non-immunoglobulin (Ig)-derived small antibody mimetics called FLuctuation-regulated Affinity Proteins (FLAPs) on the basis of our findings that immobilization of peptides in scaffolds increases binding affinity^16^. The design strategy includes selection of appropriate non-Ig small protein scaffolds using computational simulations, the computational design of target-binding peptides structurally immobilized in scaffolds, and in vitro screening methods such as phage display^16–18^. Our recent studies have identified human epidermal growth factor receptor 2 (HER2)-binding FLAPs composed of a fibronectin type III domain (FN3) scaffold. These HER2-binding FLAPs have relatively high binding affinities (dissociation constants of 24–65 nM as measured by ELISA), and we have succeeded in demonstrating not only the efficiency and simplicity of the methods, but also the target specificity and potential of FLAPs as non-Ig-derived antibody alternatives^16,17^.

In this study, we improved the method to create FLAPs by using a guide antibody (gAb) during the computational design of target-binding peptide sequences and the selection of FLAPs using a novel mammalian display system. The new method, which we have called monoclonal antibody-guided peptide identification and engineering (MAGPIE), allows us to identify high-affinity FLAPs from a small library of candidates in a single selection round. We used daclizumab, a humanized mAb against interleukin-2 receptor alpha chain (IL2Rα, CD25)^19^, as a gAb and efficiently identified CD25-binding FLAPs called Zif-FLAPs composed of target-binding peptides immobilized in the first zinc-finger domain of Zif268 (Zif), which acts as a small non-Ig protein scaffold. The successful identification of high affinity Zif-FLAPs using MAGPIE demonstrates its potential as a tool for efficient development of small non-Ig antibody mimetics using any mAb.

## Results

### Strategy for antibody-guided identification of Zif-FLAPs

**Figure 1:**
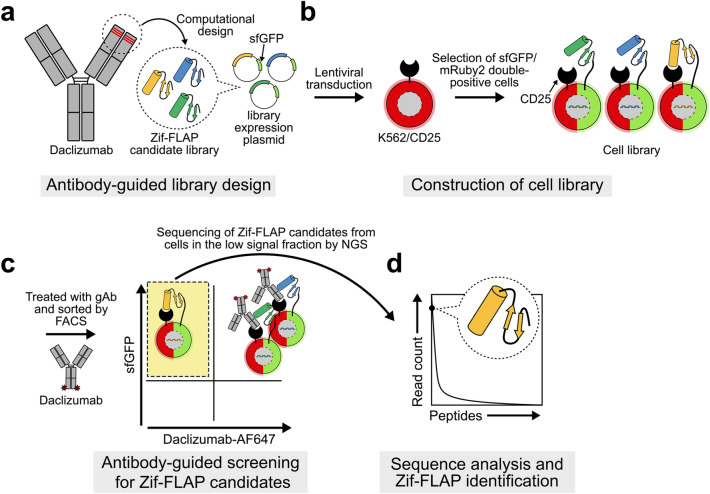
Overview of the MAGPIE system. (**a**) Computational design of the peptide library using the anti-CD25 mAb daclizumab as a guide and construction of the plasmid that expressed the Zif-FLAP candidate library. (**b**) Construction of the cell library by lentiviral transduction of K562/CD25 cells with vectors that coordinately expressed Zif-FLAP candidates and sfGFP. Linking the expression of CD25 and Zif-FLAP candidates on the cell surface with coexpression of mRuby2 and sfGFP in the cytosol, respectively, allowed visual tracking of library cells. (**c**) Antibody-guided screening of Zif-FLAP candidates using fluorescently labeled daclizumab (daclizumab-AF647). Isolation of the low signal fraction that included cells expressing high affinity Zif-FLAP candidates after incubation with daclizumab-AF647. (**d**) NGS analysis of CD25-binding sequences of the isolated Zif-FLAP candidates and identification of Zif-FLAP.

MAGPIE encompasses the entire process of developing FLAPs, as shown in Fig. 1. In the first step of MAGPIE, CD25-binding peptide sequences were selected from the complementarity-determining regions (CDRs) of the gAb, daclizumab. A cDNA library that encoded candidates of Zif-FLAPs was constructed by genetically grafting selected daclizumab CDR peptide sequences into Zif. Then, the resulting cDNA library was inserted into a plasmid that encoded superfolder GFP (sfGFP) along with other elements required to express Zif-FLAP candidates on the cell surface (Fig. 1a). The plasmid was transduced into K562/CD25 cells that expressed both CD25 and mRuby2, and sfGFP/mRuby2 double-positive cells were used as a “cell library” that coexpressed CD25 and the Zif-FLAP candidate library on the cell surface (Fig. 1b). Fluorescently labeled daclizumab was used as a gAb to screen cells with high affinity for CD25 from the cell library. Binding of high-affinity Zif-FLAPs to CD25 on the same cell surface in turn inhibited gAb binding. This allowed cells that expressed high-affinity Zif-FLAP to be collected by sorting low gAb-fluorescence signal fractions using FACS (Fig. 1c). Finally, the collected high affinity Zif-FLAP cell genome was analyzed for Zif-FLAP-coding sequences by next-generation sequencing (NGS) (Fig. 1d).

### Antibody-guided design of a Zif-FLAP candidate library

**Figure 2:**
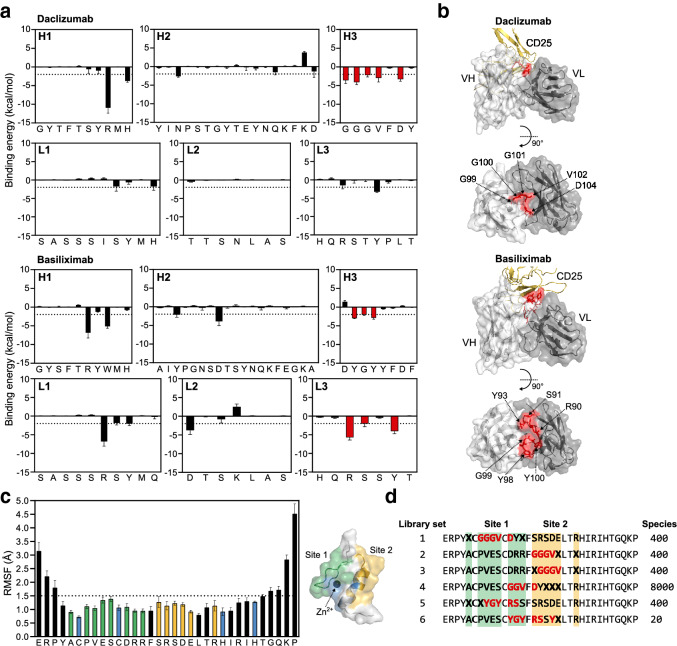
Antibody-guided design of the Zif-FLAP candidate library. (**a**) Identification of CD25-binding residues on CDRs of daclizumab and basiliximab. The binding free energy of each CDR residue to CD25 was calculated and is shown as the mean ± SEM obtained by averaging the values from the final 1 ns of three replicate 50-ns production runs. Bars in red represent residues with binding free energy values of < − 2 kcal/mol and that belonged to CDRs with three or more such residues. (**b**) Structures and molecular surface representations of the variable regions of daclizumab and basiliximab light (VL) and heavy (VH) chains. CD25 is shown in yellow, while the CD25-binding residues selected in (**a**) are depicted as the stick representation in red. (**c**) RMSF profile (left) and structure and molecular surface representation (right) of Zif. The mean ± SEM values from three replicate 100-ns production runs are shown. Bars in blue represent zinc-coordinating residues, while those in green and yellow represent residues that comprise sites 1 and 2, respectively. Sites 1 and 2 are highlighted on the structure in green and yellow, respectively. The zinc ion (black sphere) coordinated by the two cysteine and histidine residues (blue; in stick representation) is also shown. (**d**) Zif-FLAP candidate library design. The final library consisted of six library sets, four of which were designed on the basis of daclizumab, while the remaining two were designed on the basis of basiliximab. Mutated residues are shown in bold with residues in X representing those that were fully randomized and those in red representing the CD25-binding residues identified in (**a**).

The design strategy for the target-binding peptide library was based on our previous finding that immobilization of mAb-derived target binding peptides in stable protein scaffolds leads to high binding affinity^16^. First, the binding free energy values of residues that comprised the antigen-binding fragment (Fab) domains of daclizumab were calculated using the molecular mechanics energies combined with generalized Born surface area continuum solvation (MM/GBSA) method (Fig. 2a). The same calculations were performed using another anti-CD25 mAb, basiliximab, to generate additional non-daclizumab-derived candidates to evaluate the specificity of MAGPIE. Target-binding residues were then selected from the six CDRs using two criteria: a predicted binding free energy value of < − 2 kcal/mol and belonging to a CDR that contained three or more such residues. On the basis of these criteria, CD25-binding residues GGGV-D from daclizumab CDR-H3 and YGY and RS-Y from basiliximab CDRs-H3 and -L3 (Fig. 2b) were selected and used to design the target-binding peptide library.

From a pharmaceutical viewpoint, an ideal protein scaffold is small and chemically synthesizable with few or no disulfide bonds^16^. Zif is 33 amino acids long (ERPYACPVESCDRRFSRSDELTRHIRIHTGQKP) and its structure is stabilized by a zinc ion coordinated by two cysteine and two histidine residues. It has also been shown to be able to tolerate various mutations^20^. Therefore, we used this domain as the scaffold for our peptide library. The suitability of Zif as a scaffold was further confirmed by calculating the root-mean-square-fluctuation (RMSF) of the backbone atoms (C, Cα, and N) of each residue in Zif (Fig. 2c). A previously determined threshold of RMSF < 1.5 Å was used to evaluate residue fluctuation^16^. All residues in Zif except for those at the N- and C-terminal ends had RMSF values < 1.5 Å, which indicated that Zif was mostly structurally constrained and thus suitable to immobilize the fluctuation of grafted peptides.

To create the Zif-FLAP candidate library, the selected CD25-binding residues (GGGV–D, YGY, and RS–Y) were first grafted into two different sites (sites 1 and 2) of Zif (Fig. 2c,d, green and yellow). In doing so, we avoided structurally important residues, the two zinc-coordinating cysteines and histidines (Fig. 2c, blue). Then, residues in the positions indicated by “X” in sites 1 and 2 were randomized for structural optimization, which yielded six Zif-FLAP candidate library sets that contained daclizumab- or basiliximab-derived target-binding peptides. The combination of all peptides formed a Zif-FLAP candidate library (Zif lib) with a total size of approximately 1 × 10^4^ (Fig. 2d).

### Establishment of a novel mammalian cell surface display system

**Figure 3:**
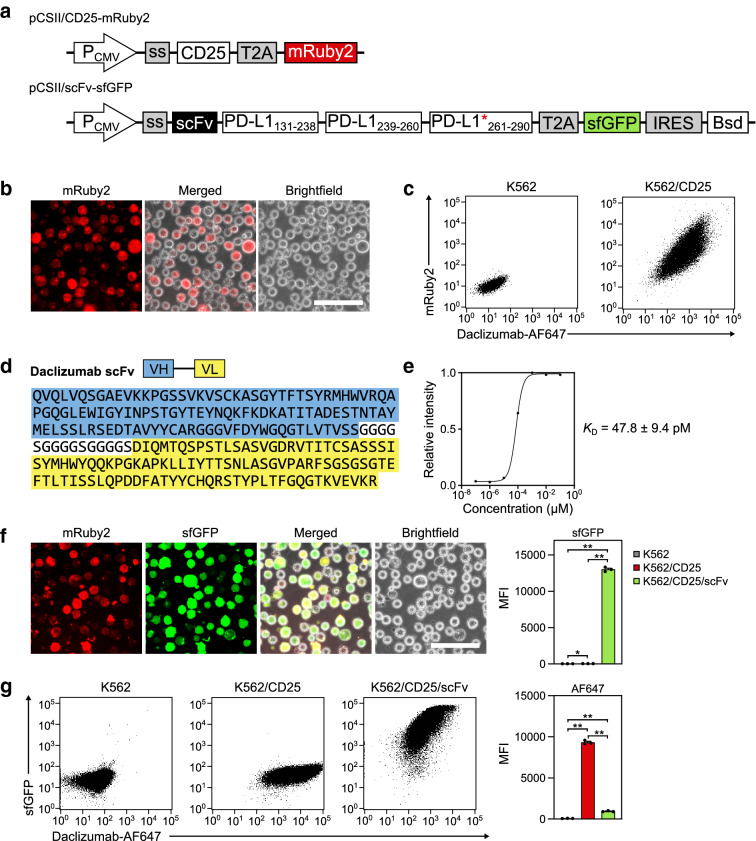
Validation of the MAGPIE system using the daclizumab scFv. (**a**) Diagram of the cDNA constructs. The red asterisk indicates that the PD-L1 sequence contained the T290M mutation. (**b**) mRuby2 expression in the K562/CD25 cell line established by lentiviral transduction with the pCSII/CD25-mRuby2 vector. Scale bar = 100 µm. (**c**) Expression of CD25 on K562/CD25 cells. Coexpression of CD25 on the cell surface with cytosolic expression of mRuby2 was detected by flow cytometry after incubation with daclizumab-AF647. Wildtype K562 cells were used as a control. (**d**) Structural diagram and sequence of the daclizumab scFv. VH and VL sequences are highlighted by blue and yellow, respectively. The linker sequence is also shown. (**e**) Estimation of daclizumab scFv binding to CD25 by an ELISA. The *K*_D_ value was estimated by curve fitting with non-linear regression and is shown with the standard error. (**f**) (left) mRuby2 and sfGFP expression in K562/CD25/scFv cells produced by lentiviral transduction with the pCSII/scFv-sfGFP vector. Scale bar = 100 µm. (right) Mean fluorescence intensity values of sfGFP calculated using flow cytometry plots from three independent experiments. *P < 0.005; **P < 0.0001. (**g**) (left) Inhibition of daclizumab-AF647 binding by surface expression of daclizumab scFv. K562/CD25/scFv cells that expressed both CD25 and daclizumab scFv were incubated with daclizumab-AF647 prior to flow cytometric analysis. Wildtype K562 and K562/CD25 cells were used as controls. Representative scatter plots from three independent experiments are shown. (Right) Mean fluorescence intensity values of AF647 calculated using flow cytometry plots from three independent experiments. **P < 0.0001.

Screening of the Zif lib using the MAGPIE system required the Zif-FLAP candidates to be coexpressed with CD25 on the same cell surface. First, we established an engineered K562 cell line, K562/CD25, which expressed the target antigen CD25 on the cell surface and mRuby2 intracellularly through lentiviral transduction of K562 cells with a plasmid, pCSII/CD25-mRuby2 (Fig. 3a, top). Fluorescence microscopy revealed that K562/CD25 cells expressed mRuby2 (Fig. 3b,c) and flow cytometric analysis of K562/CD25 cells treated with AF647-labeled daclizumab (daclizumab-AF647) confirmed cell surface expression of CD25 (Fig. 3c).

Next, to investigate whether the molecular design that expressed the Zif lib on the surface of K562/CD25 (Fig. 1b) worked as intended, we first constructed control plasmid pCSII/scFv-sfGFP that encoded a single-chain variable fragment (scFv) of daclizumab (Fig. 3d) with high binding affinity for CD25 (Fig. 3e) in place of the Zif lib (Fig. 3a, bottom). The plasmid encoded elements required to express the scFv on the cell surface: the secretion signal (1–18), extracellular domain (131–238), transmembrane domain (239–260), and intracellular domain (261–290) with T290M substitution^21^ sequences of programmed death-ligand 1 (PD-L1). cDNAs that encoded a “self-cleaving” peptide T2A^22^ and sfGFP were also added to the 3ʹ end of the PD-L1 transmembrane domain as a reporter element. pCSII/scFv-sfGFP also encoded blasticidin S deaminase as a selection element, which was cap-independently translated from an internal ribosome entry site (IRES)^23^.

After transduction of the plasmid (pCSII/scFv-sfGFP, Fig. 3a, bottom) into the K562/CD25 cell line using a lentivirus, mRuby2- and sfGFP-fluorescent cells (K562/CD25/scFv) that expressed the daclizumab scFv along with CD25 on their surface were selected (Fig. 3f). Flow cytometric analysis of K562, K562/CD25, and K562/CD25/scFv cells using daclizumab-AF647 revealed that the amount of daclizumab bound to CD25 was significantly lower in K562/CD25/scFv cells than in K562/CD25 cells (Fig. 3g), which indicated that daclizumab binding may be competitively inhibited by the scFv. These results suggested that cells expressing Zif-FLAP candidates with a high binding affinity for CD25 may reduce daclizumab-AF647 binding to CD25 on the same cell surface, and thus they could be isolated by selecting a cell population with low daclizumab-AF647 binding (i.e., the low AF647 signal fraction).

### Antibody-guided screening of Zif-FLAP candidates

**Figure 4:**
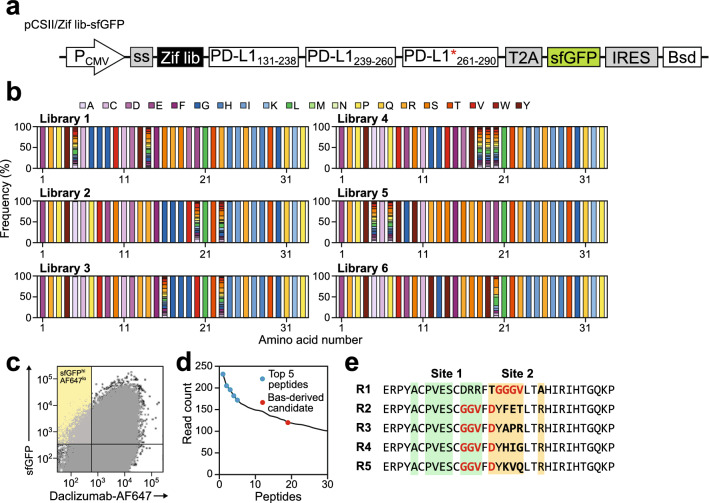
Antibody-guided screening for Zif-FLAP candidates. (**a**) Diagram of the Zif-FLAP candidate library construct. Zif lib, Zif-FLAP candidate peptide library. (**b**) Amino acid frequency in each library set prior to screening. (**c**) Isolation of the high sfGFP, low AF647 fraction after flow cytometric analysis. K562/CD25 cells transduced with the library construct were incubated with daclizumab-AF647 and then analyzed by flow cytometry for sfGFP expression and AF647 signals. The quadrant in yellow represents the fraction that was sorted out for sequencing. (**d**) Ranking of peptides in accordance with the read count after NGS analysis. The five most frequently detected sequences are shown by blue circles. The basiliximab (Bas)-derived sequence is indicated by a red circle. (**e**) Sequences of the top five peptides with the highest read counts. Residues that had been mutated are shown in bold with residues in black representing those that had been fully randomized and those in red representing the CD25-binding residues identified from daclizumab.

After confirming the functionality of antibody-guided screening using the daclizumab scFv as a positive control, we performed antibody-guided screening by constructing a Zif-FLAP candidate expression plasmid (pCSII/Zif lib-sfGFP) in which the scFv in the pCSII/scFv-sfGFP plasmid (Fig. 3a, bottom) was replaced by the Zif lib (Fig. 4a). The resulting plasmid was transduced into K562/CD25 cells by lentivirus infection and mRuby2/sfGFP-double positive cells were selected as the cell library. NGS analysis of 1 × 10^6^ cells randomly picked up from the cell library using primers specific for the Zif scaffold revealed that amino acids at “X” positions (Fig. 2d) were well randomized and that the rest of the sequences were coded as designed (Fig. 4b). After approximately 1.3 × 10^6^ library cells were incubated with daclizumab-AF647, the single cell fraction with high sfGFP and low AF647 signals (3.1 × 10^4^ cells) was sorted out (Fig. 4c). NGS analysis of the sorted cells identified 1513 unique peptides with only one of the 30 most abundant sequences from the basiliximab-guided peptide library (Fig. 4d). This result indicated that daclizumab-derived peptides were preferentially selected by daclizumab-guided screening. The top five Zif-FLAP candidates with the most reads (Zif-R1–R5) were then selected for further analysis (Fig. 4e).

### Characterization of Zif-FLAPs

**Figure 5:**
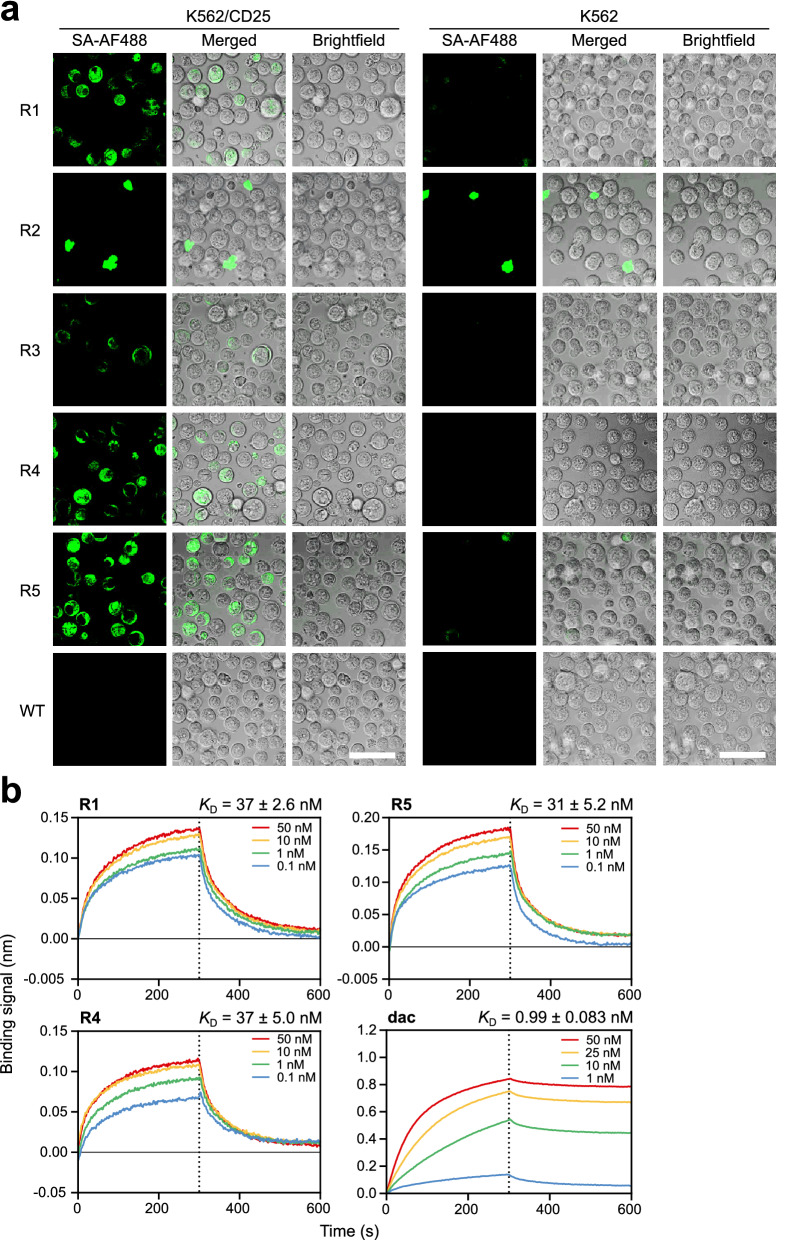
Characterization of Zif-FLAP binding to CD25. (**a**) Specific binding of Zif-FLAP candidates to K562/CD25 cells. Binding of the five peptides to fixed K562/CD25 cells was detected using SA-AF488 (green). Scale bar = 50 μm. (**b**) Binding of Zif-FLAPs and daclizumab to CD25-Fc as monitored by BLI. Biotinylated Zif-FLAPs or daclizumab (dac) was immobilized on streptavidin sensors and exposed to various concentrations of CD25-Fc. Representative sensorgrams with corresponding *K*_D_ ± SEM values calculated from three separate experiments are shown.

Zif-R1–R5 were chemically synthesized as biotinylated peptides and their CD25-binding activity was first evaluated by incubating them with K562 and K562/CD25 cell lines. Binding of the biotinylated peptides to CD25 on the cell surface was detected using AF488-conjugated streptavidin (SA-AF488) (Fig. 5a). Three peptides, Zifs-R1, -R4, and -R5, exhibited strong binding to K562/CD25 while showing little or no binding to K562 cells, which indicated that these peptides specifically bound to CD25 on the cell surface with high binding affinity. However, Zif-R2 appeared to be unstable and formed large aggregates on both K562 and K562/CD25 cells, and Zif-R3 showed weak binding to K562/CD25. Thus, Zifs-R1, -R4, and -R5 were considered as high affinity CD25-binding FLAPs and their binding kinetics to CD25 were analyzed by biolayer interferometry (BLI) (Fig. 5b, Table 1). All three peptides were found to bind CD25 with *K*_D_ values ranging from 30 to 40 nM, which are higher than that of daclizumab (~ 1 nM), but are within a clinically relevant range and may potentially elicit less “on-target off-tumor” adverse effects^24–31^.

**Table 1:** Binding kinetics of Zif-FLAPs and daclizumab to CD25.

	*K*_D_ (nM)	*k*_on_ (10^4^ M^–1^ s^–1^)	*k*_off_ (10^–2^ s^–1^)
Zif-R1	37 ± 2.6	720 ± 75	1.4 ± 0.025
Zif-R4	37 ± 5.0	1400 ± 200	1.4 ± 0.099
Zif-R5	31 ± 5.2	380 ± 6.7	1.4 ± 0.059
Daclizumab	0.99 ± 0.083	210 ± 22	0.12 ± 0.015

**Figure 6:**
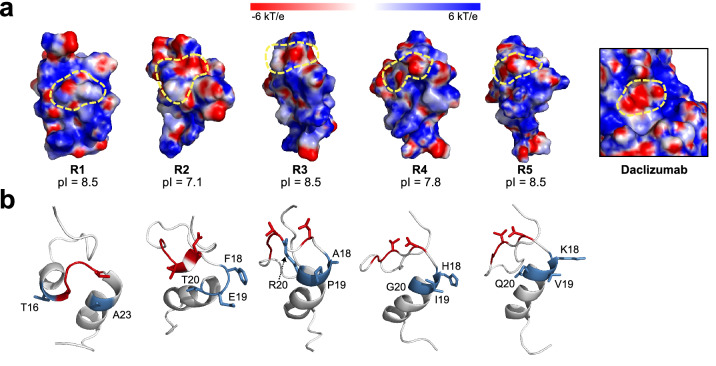
Structural analysis of Zif-R1–R5. (**a**) Electrostatic potential surfaces of Zif-R1–R5 (R1–R5) and daclizumab at pH 7.5. pI values are shown below. Dotted yellow lines indicate the location of daclizumab-derived CDR residues in each peptide. (**b**) Structures of Zif-R1–R5. Mutated residues are shown in the stick representation with daclizumab-derived residues in red and fully randomized residues in blue. Fully randomized residues are labeled in accordance with their positions.

To elucidate the molecular basis of the differences between Zif-R1–R5, we analyzed and compared the surface electrostatic potential of each of the five Zif-FLAPs. After extracting the last frame from 100-ns molecular dynamics (MD) simulations of Zif-R1–R5 and daclizumab, the surface electrostatic potential of each protein at pH 7.5 was calculated using PDB2PQR^32^ and Adaptive Poisson-Boltzmann Solver (APBS) tools^33^ (Fig. 6a). As expected from a previous study^34^, the binding surface of daclizumab was mostly positive and the GGGV–D residues used to design the Zif-FLAP candidate library created a slightly negative patch (Fig. 6a, area indicated by dotted yellow line in “Daclizumab”). The same patch was present in Zif-R1–R5 (Fig. 6a). Interestingly, the surfaces of Zif-R1 and Zif-R3–R5 appeared to be mostly positive, while that of Zif-R2 appeared to be largely negative. Calculation of the isoelectric point (pI) using the European Molecular Biology Open Software Suite (EMBOSS)^35^ for each of the peptides confirmed this observation with Zif-R2 having the lowest pI of the five at 7.1 (Fig. 6a), which may explain its tendency to aggregate in solution (Fig. 5a). Moreover, the weaker binding of Zif-R3 (Fig. 5a) may be due to the presence of a highly positively charged arginine residue (Fig. 6b), which may have been influenced by the proximity of the negatively charged cell membrane while screening. As a result, Zif-R3 may have adopted a conformation with higher affinity for CD25 on the cell surface.

## Discussion

This study demonstrates the effectiveness of the MAGPIE system to identify small antibody mimetics such as Zif-FLAPs. Using MAGPIE, we identified three Zif-FLAPs, Zifs-R1, -R4, and -R5, which bound CD25 with high specificity and affinity within only one round of screening using a small library of approximately 1 × 10^4^. As a mammalian display system, MAGPIE has several advantages over other cell display systems and phage display. MAGPIE makes it possible to screen binding peptides targeting membrane proteins that are natively folded on the cell surface. Additionally, by anchoring target-binding peptides on the cell surface and selecting high binders on a single cell basis, our MAGPIE system has an advantage over the secretory peptide system by minimizing the occurrence of false positives arising from secreted peptides that bind to neighboring cells. Furthermore, by performing computational design on the basis of the structure of mAbs, MAGPIE provides a library that is small in size, but has sufficient content for screening. This overcomes a major issue in mammalian display systems, in which low transfection efficiencies often limit the sizes of libraries that can be screened, typically up to diversities of only ~ 10^7^ variants^12^. Using antibody-guided design, we were able to pinpoint specific residues to mutate and apply saturation mutagenesis to only two to three sites per library set, which allowed us to minimize the overall size of the library. Applying saturation mutagenesis to all mutation sites would have resulted in an overall library size of ~ 4 × 10^9^, the screening of which would not have been feasible with a mammalian display system without compromising library representation.

MAGPIE was developed to improve the previous methods of FLAP identification^16–18^. In this study, we identified several areas that need further improvement. First, we need to control or measure the number of molecules (both target proteins and target-binding peptides) expressed on the cell surface to more accurately assess the binding of a gAb to the cell library. Optimizing the conditions for selecting cell fractions expressing target-binding peptides that inhibit gAb binding is crucial to identify high binders. One approach to achieve this is to adopt index sorting technology^36^ to simultaneously evaluate the fluorescence intensities of the labeled gAb, mRuby2, and sfGFP. These correlate with gAb binding inhibition, target protein expression levels, and library protein expression levels, respectively. Second, although only one round of screening was performed in this study, it may be interesting to determine how the population of sorted peptides changes over several rounds of screening.

A difference between the current study and those preceding it is the scaffold protein. The primary scaffold protein used in our previous studies was the FN3 protein, the loops of which closely resemble those of CDRs^16–18^. On the other hand, the scaffold protein used in the current study, Zif, is made up of an α-helix and β-sheet stabilized by a zinc ion^20^ and thus does not have long loop regions that bear a close resemblance to those of CDRs. Therefore, in this study, on top of choosing to graft individual CDR residues instead of peptides, we decided to further mutate several residues surrounding those grafted from the CDR to optimize the binding structure.

Although mammalian display systems have been used to identify non-antibody target-binding peptides^8,9^, MAGPIE is the first screening system to incorporate the use of a mAb as a guide for the design and selection of small antibody mimetics. While this may limit the use of MAGPIE to screening peptides that target molecules for which mAbs have already been developed, it also opens up the possibility of using MAGPIE to efficiently select peptides with specific epitopes by simply changing the gAb. This is especially important when targeting molecules such as CD25 that is expressed on both regulatory (Treg) and effector T (Teff) cells. Recently, the development of mAbs optimized to target Treg cells while preserving signaling on Teff cells has been described^37^. Using MAGPIE, it may be possible to develop target-binding peptides that share the same epitope with such mAbs. Alternatively, MAGPIE may be also used to efficiently identify binding peptides for a specific molecule using a protein that binds to the specific molecule as a guide instead of a mAb.

Although Zif-R2 was selected as a high affinity binder to CD25 when it was expressed on the cell surface (Fig. 4d), synthesized Zif-R2 formed large aggregates in PBS (Fig. 5a). The sequence of Zif-R2 differs from those of Zif-R4 and Zif-R5 by only three amino acid residues (Fig. 4e). Although it is currently difficult to identify and completely eliminate aggregation-prone peptides such as Zif-R2 when screening with molecular display systems, it may be possible to reduce the occurrence of such peptides by further refining the initial library design using an algorithm that predicts protein aggregation propensity^38^. Additionally, in silico redesign has successfully been used to improve the stability of target-binding proteins in solution^39^.

The Zif-FLAPs identified and characterized in this study had *K*_D_ values of 30–40 nM. Their affinities were more than ten times weaker than that of daclizumab, which had a *K*_D_ value of 0.99 nM (Fig. 5b), largely due to the slower off-rates. This may be due to the difference in binding modes between daclizumab (bivalent) and the Zif-FLAPs (monovalent). Using a monovalent antibody fragment (e.g., scFv, Fab) as opposed to whole antibody to evaluate the binding of the Zif-FLAPs might provide a better comparison. Alternatively, the low binding affinities of Zif-FLAPs may also be due to high *k*_off_ values (Table 1), which in turn translated to faster dissociation. However, it is important to again note that the Zif-FLAPs were identified after only one round of selection and that they did not undergo any affinity maturation. Therefore, it may be possible to improve their binding affinity to CD25. The fact that Zif-R4 and Zif-R5, but not Zif-R3, contain basic residues (H and K, respectively) and aliphatic residues (I and V, respectively) in the same positions (Fig. 4e) suggests that such residues play an important role in the binding of Zif-FLAPs to CD25. This observation may provide a starting point to improve the dissociation rates and overall binding affinities of future FLAPs. Additionally, there have been several reports, mostly with chimeric antigen receptor T cells, in which lower affinities (with *K*_D_ values ranging from 20 to 320 nM) were shown to yield better safety profiles^24–31^.

Here, we describe the proof-of-concept for our novel peptide screening system, MAGPIE. MAGPIE is a highly versatile system that can be applied to a wide range of targets and proteins, and may be indispensable for efficient development of alternatives to current antibody drugs.

## Methods

### MD simulations

The initial coordinates of the daclizumab Fab-CD25 and basiliximab Fab-CD25 complexes were obtained from PDB accession codes 3NFP and 3IU3, respectively, whereas those of Zif were obtained from 4R2A (amino acid numbers 335–367). All MD simulations were performed using the AMBER 16 program package^40^ on TSUBAME (Global Scientific Information and Computing Center, Tokyo Institute of Technology).

The binding free energy calculations using the daclizumab Fab-CD25 and basiliximab Fab-CD25 complexes were performed as described previously^18^. Briefly, the systems were fully solvated with explicit solvent with counterions added to obtain electrostatic neutrality. The AMBER ff14SB force field for proteins and the TIP3P model for water molecules were used for the simulations. Optimization of the systems by energy minimization was followed by equilibration with backbone restraints. Production runs were performed for a total of 50 ns, from which the final 1 ns was used for binding free energy calculations with the MM/GBSA module.

To analyze the backbone (C, Cα, and N atoms) fluctuation of each residue in Zif, Amber ff14SB force fields and the GB/SA implicit solvent model were used. Then, 100-ns production runs were performed as described previously^18^. The final 5 ns (i.e., 95–100 ns) were used to calculate RMSF values with the cpptraj module.

To predict the structures and dynamics of Zif-R1–R5, production runs were performed for 100 ns for trajectory analysis. The AMBER ff14SB force field and GB/SA implicit solvent model were used. The time-step for MD simulations was set to 2 fs with the SHAKE algorithm. A non-bonded cutoff of 999.9 Å was used. The temperature was kept constant at 300 K using the Berendsen rescaling method. Electrostatic potential at pH 7.5 was calculated using PDB2PQR^32^ and APBS^33^ tools with the last frame from each simulation.

### Plasmid construction

All recombinant DNA experiments were approved by the Recombinant DNA Experimental Safety Management Committee of the Tokyo Institute of Technology.

To express CD25, cDNA that encoded a fusion protein, which consisted of human CD25, a “self-cleaving” T2A peptide (EGRGSLLTCGDVEENPGP), and mRuby2, was inserted into the multiple cloning site of the CSII-CMV-MCS vector (RIKEN Bio-Resource Center, Ibaraki, Japan). The resulting vector was named pCSII/CD25-mRuby2.

To display proteins and peptides on the mammalian cell surface, fusion proteins that consisted of the PD-L1 secretion signal (ss; amino acid numbers 1–18), proteins and peptides to be displayed, and the truncated T290M PD-L1 mutant (amino acid numbers 131–290) were designed (UniProt: Q9NZQ7). Then, cDNAs that encoded (i) the fusion protein that consisted of the PD-L1 ss, daclizumab scFv, a linker sequence (GEQKLISEEDGHHHHHHGGGGSGGGGS), the truncated T290M PD-L1 mutant, T2A peptide, and sfGFP and (ii) the fusion protein, which consisted of the PD-L1 ss, Zif peptide library, a linker sequence, the truncated T290M PD-L1 mutant, T2A peptide, and sfGFP, were inserted into the multiple cloning site of the CSII-CMV-MCS-IRES2-Bsd vector (RIKEN Bio-Resource Center). The Zif peptide library consisted of six library sets, ERPYXCGGGVCDYXFSRSDELTRHIRIHTGQKP (400 sequences), ERPYACPVESCDRRFGGGVXLTXHIRIHTGQKP (400 sequences), ERPYACPVESCDRRFXGGGVLTXHIRIHTGQKP (400 sequences), ERPYACPVESCGGVFDYXXXLTRHIRIHTGQKP (8000 sequences), ERPYXCXYGYCRSSFSRSDELTRHIRIHTGQKP (400 sequences), and ERPYACPVESCYGYFRSSYXLTRHIRIHTGQKP (20 sequences), where X represents randomized amino acids encoded by NNK codons. The frequency of occurrence of each sequence was adjusted to be the same. The resulting vectors were named pCSII/scFv-sfGFP and pCSII/Zif lib-sfGFP, respectively.

To purify the daclizumab scFv, cDNA that encoded the fusion protein, which consisted of the PD-L1 secretion signal, daclizumab scFv, and His6 peptide tag, was inserted into the pcDNA3.1 vector (Thermo Fisher Scientific, Waltham, MA, USA). The resulting vector was named pcDNA/ss-scFv.

### Cell lines

The Lenti-X 293 T cell line, which is a subclone of the human embryonic kidney cell line HEK 293, human cervical cancer cell line HeLa, and human chronic myelogenous leukemia cell line K562 were obtained from Clontech (Mountain View, CA, USA), RIKEN Bio-Resource Center, and JCRB Cell Bank (Osaka, Japan), respectively. K562/CD25 and K562/CD25/scFv cell lines were established by lentiviral transduction of K562 and K562/CD25 cells with pCSII/CD25-mRuby2 and pCSII/scFv-sfGFP vectors, respectively, followed by cell cloning through serial dilution.

Lenti-X 293 T cells, and K562, K562/CD25, and K562/CD25/scFv cells were maintained at 37 °C with 5% CO_2_ in 10% FBS-DMEM (Nacalai Tesque, Kyoto, Japan) and 10% FBS-RPMI-1640 (Invitrogen, Carlsbad, CA, USA), respectively. All media were supplemented with penicillin (100 U/mL) and streptomycin (100 µg/mL) (Nacalai Tesque). Cells were regularly checked for mycoplasma contamination using a mycoplasma test kit (Lonza, Basel, Switzerland).

### ELISA

HeLa cells (1 × 10^6^ cells in 10-cm dish) were transfected with the pcDNA/ss-scFv vector using PEI. After incubation in 0.5% FBS-DMEM for 24 h, the secreted His6-tagged scFv protein in the culture medium was purified using a HisTrap HP column (Cytiva, Marlborough, MA, USA) with the AKTA pure 25 system (Cytiva).

ELISAs were carried out in 96-well black plates (Thermo Fisher Scientific) with all steps performed at room temperature with the exception of coating that was conducted by incubation with recombinant human CD25-Fc (R&D systems, Minneapolis, MN, USA) overnight at 4 °C. Blocking was performed with 2% Perfect Block (MoBiTec) in PBS (PBS-PB) for 2 h, followed by washing three times with PBS-T and incubation with His6-tagged scFv in PBS-PB for 1 h. The wells were then washed again three times with PBS-T and incubated with a 1000-fold diluted HRP-conjugated α-His tag antibody (Abcam, Cambridge, MA, USA) in PBS-PB for 1 h. Before detection using a QuantaRed Enhanced Chemifluorescent HRP Substrate kit (Thermo Fisher Scientific), the wells were washed three times with PBS-T and then another three times with PBS. The resulting fluorescence signals were measured using an Infinite F500 plate reader (Tecan, Mannedorf, Switzerland) with specific filters (Ex/Em = 535 nm/590 nm).

### Lentiviral transduction

To produce recombinant lentiviruses, Lenti-X 293 T cells were first cultured to 70%–80% confluence in a 10-cm dish. pCSII/CD25-mRuby2, pCSII/scFv-sfGFP, and pCSII/Zif lib-sfGFP vectors were combined with Lentiviral High Titer Packaging Mix (Takara Bio, Shiga, Japan) and then transfected into Lenti-X 293 T cells with Lipofectamine LTX (Invitrogen) in accordance with the manufacturer’s instructions. After 24 h of incubation at 37 °C with 5% CO_2_, the medium was replaced and 10 μM forskolin (Fujifilm Wako Pure Chemical, Osaka, Japan) was added. After incubation for 48 h, the lentivirus in the medium was collected, filtered through a 0.45-μm membrane, and concentrated using a Lenti-X Concentrator (Clontech) in accordance with the manufacturer’s instructions.

A modified spinoculation technique was used to infect target cells with the lentivirus^41^. For library screening, K562/CD25 cells were transduced at a multiplicity of infection (MOI) of 0.3 to produce a population of cells with one gene integration per cell. First, the cells were incubated in medium with the virus stock and 8 μg/mL polybrene (Nacalai Tesque). The mixture was then spun down in a swinging bucket centrifuge at 800×*g* for 90 min at 20 °C. Then, the cells were resuspended through gentle pipetting and transferred to culture dishes. After incubation for 24–48 h at 37 °C with 5% CO_2_, the transduction efficiency was verified under a Biorevo BZ-X710 fluorescence microscope (Keyence, Osaka, Japan) with the appropriate filters (Ex/Em = 470 ± 40 nm/520 ± 50 nm for sfGFP and 545 ± 25 nm/605 ± 70 nm for mRuby2) or using an iCyt ec800 flow cytometer (Sony Biotechnology, San Jose, CA, USA).

### Cell sorting

To generate K562/CD25/Zif lib cells, 7.2 × 10^6^ K562/CD25 cells were lentivirally transduced with the pCSII/Zif lib-sfGFP vector at an MOI of 0.3, followed by selection with blasticidin (Bsd; 5 μg/mL) for 3 days. To prepare for cell sorting, 1.3 × 10^6^ Bsd-resistant cells were resuspended in 3% FBS-PBS and incubated with 1000-fold-diluted AF647-conjugated daclizumab (#FAB9927R, R&D Systems) for 1 h at 4 °C. The AF647-labeled K562/CD25/Zif lib cells were then sorted with a FACSAria III fluorescence activated cell sorter (BD Biosciences, San Jose, CA, USA) based on GFP and AF647 signal intensity.

### Next-generation sequencing

Genomic DNA from the collected cell fraction was extracted using a GenElute Mammalian Genomic DNA Miniprep Kit (Sigma-Aldrich, St. Louis, MO, USA) in accordance with the manufacturer’s instructions. DNA fragments that contained the sequences of the Zif mutants were amplified using a Kapa HiFi HotStart ReadyMix PCR Kit (Kapa Biosystems, Wilmington, MA, USA) and purified using AMPure XP beads (Beckman Coulter Life Sciences, Indianapolis, IN, USA) in accordance with the manufacturers’ instructions. Sequencing was performed on the Illumina MiSeq platform by Macrogen Japan Corp. (Tokyo, Japan) with a read depth of 111,000 reads. Sequences were ranked in accordance with the read count.

### Cell staining and confocal microscopy

To characterize binding of the Zif-FLAP candidates, K562 or K562/CD25 cells were first adhered to the glass bottom of a tissue culture-treated cell imaging dish (Eppendorf, Hamburg, Germany) by gravity sedimentation^42^. Briefly, the cells were washed twice with TBS and resuspended to 1 × 10^6^ cells/ml in TBS. Then, 400 μL of the cell suspension was pipetted onto the dish and incubated for 30 min at room temperature. The medium was aspirated and the cells were fixed in 4% paraformaldehyde at room temperature for 10 min and then treated with blocking solution (1% BSA in TBS) at room temperature for 1.5 h. Zif-FLAP candidates, which were biotinylated along with their chemical synthesis by a commercial facility (Biologica, Nagoya, Japan), were incubated in a zinc ion-containing buffer (0.5 mM DTT and 100 μM ZnCl_2_ in TBS) at room temperature for approximately 5 min to ensure proper folding before dilution to a final concentration of 1 μM with TBS. The cells were then incubated with the Zif-FLAP candidates at 4 °C for 16 h. Binding was detected by first incubating the cells with 5 μg/mL AF488-conjugated streptavidin (Invitrogen) at 4 °C for 30 min. After replacement with TBS buffer, the cells were visualized under a laser scanning confocal microscope (Zeiss LSM 780, Zeiss, Jena, Germany) using a 488-nm argon laser.

### Biolayer interferometry

Binding kinetics data were obtained using a ForteBio Octet K2 instrument (Sartorius, Göttingen, Germany). The assays were performed at 30 °C in 96-well black plates. Biotinylation of daclizumab (#MAB9927, R&D Systems) was performed using a biotin labeling kit (Dojindo, Kumamoto, Japan) in accordance with the manufacturer’s instructions. Biotinylated daclizumab or Zif-FLAP were loaded onto the surface of a streptavidin biosensor (Sartorius) at 100 nM in kinetic buffer (0.1% BSA and 0.002% Tween-20 in TBS, pH 7.5) for 300 s. After washing (30 s) and equilibrating (60 s) the biosensor, the association of the ligand on the biosensor to CD25-Fc (ACROBiosystems, Newark, DE, USA) was measured for 300 s. The biosensor was then dipped back into buffer for another 300 s to measure dissociation. Systematic baseline drift correction was conducted by subtracting the shift recorded for sensors loaded with ligand, but incubated without analyte. Data analysis and curve fitting were performed using Octet software version 11.0. Experimental data were fitted with the binding equations available for a 1:1 interaction with local fitting to calculate *K*_D_, *k*_on_, and *k*_off_ values using data obtained from four analyte (CD25-Fc) concentrations.

### Statistical analyses

All experimental data were acquired three times and used for statistical analysis. GraphPad Prism ver. 9.1.2 (GraphPad Software) was used for statistical calculations. For unpaired *t*-tests, *P* < 0.05 was considered statistically significant.
